# Severe and long-lasting alteration of albumin redox state by plasmapheresis

**DOI:** 10.1038/s41598-022-16452-4

**Published:** 2022-07-16

**Authors:** Kristina Boss, Mark Stettner, Fabian Szepanowski, Anne K. Mausberg, Margret Paar, Refik Pul, Christoph Kleinschnitz, Karl Oettl, Andreas Kribben

**Affiliations:** 1 Department of Nephrology, University Hospital Essen, University Duisburg-Essen, 45147 Essen, Germany; 2 Department of Neurology and Center for Translational Neuro- and Behavioural Sciences (C-TNBS), University Hospital Essen, University Duisburg-Essen, Essen, Germany; 3 Division of Physiological Chemistry, Otto Loewi Research Center, Medical University of Graz, Graz, Austria

**Keywords:** Nephrology, Neurology

## Abstract

Plasmapheresis (PE) is an established form of therapeutic apheresis (TA). Purpose of this longitudinal prospective single center study was to investigate the effect of PE on albumin redox state (ARS), as infusion of commercial albumin during PE may alter albumin oxidation which has an impact on its functional properties and oxidative stress level. 43 subjects with autoimmune-mediated neurological disorders were included. 20 subjects in the experimental group received five treatments of PE. 13 subjects received five treatments of immunoadsorption and 10 subjects received no TA as controls. ARS was determined before and after TA and 12 days after the last TA by fractionating it into human mercaptalbumin (HMA), human non-mercaptalbumin 1 (HNA-1), and human non-mercaptalbumin 2 (HNA-2) by high-performance liquid chromatography. Irreversibly oxidised HNA-2 increased over the course of five PE treatments from 2.8% (IQR 1.3–3.7%) to 13.6% (IQR 10.9–15.9) (*P* < 0.01) and remained elevated 12 days after the last PE procedure (7.7% IQR 7.1–10.5, *P* < 0.05). The study showed for the first time that PE exerts a severe and long-lasting alteration on ARS indicating a new adverse effect of PE, that may influence oxidative stress level.

## Introduction

Therapeutic apheresis (TA) is an umbrella term for several methods for the extracorporeal elimination of pathogenic proteins, protein-bound pathogens or cells from the blood. The two most common procedures are plasmapheresis (PE) and immunoadsorption (IA), but TA also includes e.g. photopheresis and leukapheresis^1^. Plasmapheresis is used as a treatment option for more than 80 serious diseases including several nephrological diseases, such as thrombotic microangiopathy and indications in the field of kidney transplantation but is also an important treatment option in neurological diseases, such as immune-mediated neuropathies^2–4^. So, PE constitutes the standard therapy for steroid-refractory relapse in multiple sclerosis and clinically isolated syndrome. However, IA has evolved into an alternative approach to plasmapheresis^5,6^.

Both methods of therapeutic apheresis remove pathological blood components such as immunoglobulins, inflammatory cytokines, and complement factors. In both processes, plasma is separated first and then transferred to a secondary circuit, but the following steps differ between PE and IA. On the one hand, in PE, the plasma is completely discarded and replaced isovolaemicly by a solution of commercial albumin and dialysate. On the other hand, in IA, pathological blood components are eliminated selectively by immunological or physicochemical adsorption or precipitation. Therefore, in IA, the patient´s plasma passes through an adsorber without substitution of albumin. In both methods cellular components are then reinfused to the patients, in the case of IA together with the purified plasma, in the case of PE with the commercial albumin solution^1^.

Both methods have a similar risk for catheter-thrombosis or catheter-infection, as both methods need the same vascular access^7^. Also, side effects due to the extracorporeal circuit, as for example nausea, shivering, or muscle cramps are equally frequent^8^. But other side effects differ between PE and IA: During PE, substituting plasma with commercial albumin is known to have several disadvantages, in particular reduction of plasmatic anticoagulation, allergic reactions, and antibody deficiency condition, which could lead to severe infections^8^. It is reported, that patients with neurological diseases receiving PE have an overall higher risk of side effects compared to patients with nephrological diseases receiving PE^8^. On the other hand, IA is associated with overall fewer adverse effects due to the selective elimination of pathological blood components and most of the adverse effects could be considered as tolerable after timely treatment^9^. But there are disadvantages of this method as well: it causes a decrease in blood pressure in more than 10% of patients and cannot be administered to patients being treated with angiotensin-converting enzyme (ACE) inhibitors due to its interaction with the adsorber material and consecutively the risk of bradykinin-mediated anaphylactic reaction^1^.

Commercial albumin is known to be different from endogenous albumin in terms of its redox state and its oxidative modification has an impact on its functional properties^10–12^. Thus, with infusing large amounts of commercial albumin, it could be assumed that changes occur in both the quantity and the quality of the patient’s albumin. Albumin is necessary not only for maintaining oncotic pressure and serving as a transport protein for a variety of substances, such as fatty acids, hormones, and drugs, but also for acting as an antioxidant^13–15^. This characteristic also makes it a suitable target structure for assessing the degree of oxidative stress by evaluation of the albumin redox state.

Albumin redox state may be defined in different terms. Here we understand it as the redox state based on cysteine-34 (Cys-34) which may occur (i) in the fully reduced form as thiol, human mercaptalbumin (HMA), (ii) in a mild reversibly oxidized form as a disulfide mainly with another cysteine, human nonmercaptalbumin-1 (HNA-1) and (iii) irreversibly oxidized to the sulfinic or sulfonic acid form, human nonmercaptalbumin-2 (HNA-2). The degree of albumin oxidation and especially the proportion of irreversibly oxidized albumin (HNA-2) has impact on the functional properties and the oxidative stress level^10^.

In summary, commercial albumin is often used, but little is known about the influence of albumin substitution on albumin quantity and albumin redox state over the course of therapeutic apheresis. Here we describe a new adverse effect of PE on albumin redox state for patients without kidney or liver impairment. Patients undergoing IA and patients not undergoing therapeutic apheresis served as control subjects.

## Methods

### Study design and patient enrolment

The study was conducted in the Department of Nephrology and the Department of Neurology of the University Hospital Essen, Essen, Germany, from November 2019 to July 2021. To evaluate the effect of plasmapheresis on the quantity and the redox state of albumin in patients without kidney or liver impairment, the study included patients with inflammatory autoimmune diseases who required an inpatient stay at the University Hospital Essen, Essen, Germany. Acute and chronic liver impairment were defined according to the Child–Pugh definition and the Kings College Hospital Criteria^16,17^, acute kidney injury or a chronic kidney disease were defined according to Kidney Disease: Improving Global Outcomes (KDIGO) definitions^18,19^. All patients included in the study had an autoimmune mediated condition[either multiple sclerosis, optic neuritis, neuromyelitis optica spectrum disorder (NMOSD), Guillain–Barré syndrome (GBS), or a chronic inflammatory demyelinating polyneuropathy (CIDP)], and were treated either with five courses of therapeutic apheresis (either PE or IA) or were not treated (non-treatment group). Patients in the non-treatment group were enrolled in the study during a required outpatient visit at the Department of Neurology at the University Hospital Essen, Essen, Germany. Inclusion criteria were age ≥ 18 years, ability to provide informed consent, and existing demyelinating disease. Exclusion criteria were age ≤ 18 years, no declaration of consent, and no existing demyelinating disease. Patients were assigned to one of the two treatment groups (PE or IA). The indication for therapeutic apheresis and choice of treatment mode (PE or IA) were independent from the study and made according to medical aspects without randomization. Patients who have received IA or no TA served both as controls.

All patients enrolled in the study underwent baseline laboratory studies. Albumin quantity and albumin fractions were measured in the non-treatment group upon study enrolment and in the treatment group at eight time points: before and after the first, the third, and the fifth apheresis procedure, and 24 h after the first apheresis procedure. Additionally, albumin quantity and albumin redox state were measured 12 days after the fifth apheresis procedure as a long-term follow up. The commercial albumin used for PE substitution was also analysed with regard to albumin redox state.

### Plasmapheresis and immunoadsorption

Plasmapheresis was carried out according to the German Apheresis Standard^1^. PE volume was determined by each patient’s weight and haematocrit. Commercial human albumin 20% (Plasbumin 20, Grifols, Barcelona, Spain) was diluted with Duosol 4 K (B Braun, Melsungen, Germany) to a 4% plasma substitution solution. PE was performed with a Spectra Optia apheresis system (Terumo, Shibuja, Japan) via central vein access. All patients in this study group underwent five PE treatments. As a precautionary measure to prevent bleeding due to modulation of the plasmatic coagulation system, fibrinogen was measured daily and PE was performed only when the patient’s fibrinogen level was higher than 150 mg/dl. If the fibrinogen level was below 150 mg/dl, plasmapheresis was postponed to the following day and the treatment was then performed if a further measurement detected a fibrinogen level > 150 mg/dl. The aimed PE interval was one procedure every second day or rather five treatments within ten days.

Immunoadsorption (IA) was also carried out according to the German Apheresis Standard^1^. All patients in this study group underwent five IA treatments. The aimed IA interval was one procedure every second day or rather five treatments within ten days. IA was performed with an Octo Nova adsorption system with TR-350 Tryptophan adsorber (Diamed, Cressier, Switzerland) with a 2-l treatment volume and a 20-ml/h plasma flow.

### Baseline laboratory measurements

To exclude relevant confounding conditions, baseline laboratory measurements were performed. None of the patients had an acute kidney injury or a chronic kidney disease according to Kidney Disease: Improving Global Outcomes (KDIGO) definitions^18,19^, nor did any patient have an acute or chronic liver impairment according to the Child–Pugh definition and the Kings College Hospital Criteria^16,17^. INR was ≤ 1.0 in all subjects. Variables associated with systemic infection (white blood cell count, C-reactive protein) were only marginally elevated compared to the normal range of the parameter for healthy subjects.

Baseline laboratory measurements were performed by Central Laboratory of University Hospital Essen, Essen, Germany. Creatinine was determined via Jaffe-method. Albumin quantity was determined using Albumin Bromocresol Green (BCG) Assay Kit (Abcam) by UV–Vis spectrometry.

### High-performance liquid chromatography

The redox state of plasma albumin was determined by fractionating it into human mercaptalbumin (HMA), human nonmercaptalbumin-1 (HNA-1), and human non-mercaptalbumin-2 (HNA-2) with high-performance liquid chromatography (HPLC), as described by Oettl et al.^10^. In healthy humans, albumin predominantly (70–80%) circulates in a reduced state (HMA), with a free thiol group in the Cys-34 residue acting as a free radical scavenger for reactive oxygen and nitrogen species. Also, lower amounts of HNA-1 (20–30%), the reversibly oxidized state of albumin, and HNA-2, the irreversibly oxidized state, circulate in the blood (2–5%)^10^. Interpretations of the results of this study refers to these albumin redox state proportions. No measurements of the proportion of albumin redox state in healthy subjects were carried out in the framework of this study.

### Statistical analysis

All statistical analyses and graphical evaluations were performed with GraphPad Prism software (version 9.1; San Diego, CA, USA). A two-way ANOVA has been performed to determine statistically significant differences between the means of the different groups (PE and IA) during the treatment as a whole. The Kolmogorov–Smirnov test excluded normality of some data sets. In these cases, the Wilcoxon matched-pairs signed rank test was used for comparison of medians; otherwise, a paired-t test was used. Statistical significance was set at the level of *P* < 0.05.

### Ethics approval

The study was performed in accordance with the Declaration of Helsinki and the International Conference on Harmonization Good Clinical Practice guidelines. The study was approved by the local ethics committee of the University of Duisburg-Essen (18-8227-BO). All patients gave written informed consent for the study.

### Consent for publication

All patients gave written informed consent to the study.

### Institutional review board statement

The study was conducted according to the guidelines of the Declaration of Helsinki, and approved by the local ethics committee of the University of Duisburg-Essen (18-8227-BO).

## Results

### Patient and treatment characteristics

A total of 43 patients participated in this study. Twenty of the 43 (47%) were men and 23 (53%) were women. The average age was 46 years (range, 20–79 years). The most common disease in the study population was multiple sclerosis (26 of 43 patients, 60%). The treatment group consisted of 20 patients who underwent PE. The group of control subjects consisted of 23 patients (13 patients with IA and 10 patients with no treatment). Detailed baseline laboratory findings for treatment subjects and control subjects are presented in Table 1 and in the supplement Table 2. Detailed patient characteristics are presented in Table 2.

**Table 1:** Baseline laboratory measurements.

Baseline laboratory measurement	Treatment subjects	Control subjects	
	Plasmapheresis	Immunoadsorption	Non-treatment group
	n = 20	n = 13	n = 10
White blood cell count (× 10^9^/l) (normal, 3.6–9.2)	8.5 5.4–10.4	7.9 4.9–9.7	5.7 4.2–7.7
Haemoglobin (g/dl) (normal, 13.7–17.7)	13.4 12.5–15.5	13.4 11.9–13.7	15.2 13.4–16.0
Haematocrit (l/l) (normal, 0.4–0.5)	0.4 0.3–0.4	0.4 0.3–0.4	0.4 0.4–0.5
Platelets (× 10^9^/l) (normal, 140–320)	275 234–302	255 203–295	240 193–277
Albumin (g/dl) (normal 3.4–4.8)	4.1 3.7–4.3	3.9 3.6–4.1	4.5 4.0–4.6
C-reactive protein (mg/dl) (normal, < 0.5)	0 0–0.5	0 0	0 0
Fibrinogen (mg/dl) (normal, 188–384)	355 154–465	320 197–357	–

Table shows median and interquartile range (IQR).

**Table 2:** Patient and treatment characteristics.

Characteristic	Treatment subjects	Control subjects	
	Plasmapheresis	Immunoadsorption	Non-treatment group
	n = 20	n = 13	n = 10
**Demographic data**			
Age (years), mean (range)	48 (20–79)	51 (25–74)	37 (21–60)
Men, n (%)	10 (50%)	4 (31%)	6 (60%)
Women, n (%)	10 (50%)	9 (69%)	4 (40%)
**Demyelinating disease, n**			
Multiple sclerosis	11	5	10
Optic neuritis	1	2	0
NMOSD	3	3	0
CIDP	3	2	0
GBS	2	1	0
Total treatment period (d), mean (range)	10.3 (8–14)	9.6 (6–11)	–
Treatment volume (l), mean (range)	2.8 (2.0–3.5)	2.0 (2.0–2.0)	–

*NMOSD* Neuromyelitis optica spectrum disorder, *CIDP* Chronic inflammatory demyelinating polyneuropathy, *GBS* Guillain–Barré syndrome, *d* Days, *l* Liter.

The entire PE or IA therapy (five treatments each) lasted for a mean of 10 days. The mean PE volume was 2.8 l per treatment, whereas the mean IA volume was 2.0 l per treatment.

### Albumin quantity

Albumin quantity was determined before and after the first, third, and fifth therapeutic apheresis and additionally 24 h after the first treatment. In both the treatment group (PE) and in the control groups (IA and non-treatment), the baseline albumin was within the normal range (3.4–4.8 g/dl). None of the patients showed a hypoalbuminemia. In patients receiving PE, the albumin level remained largely constant over the course of the five treatments (Table 3). A slight increase was observed only after the first plasmapheresis.

**Table 3:** Albumin quantity.

	Treatment subjects	Control subjects	
	Plasmapheresis	Immunoadsorption	Non-treatment group
	n = 20	n = 13	n = 10
Pre 1. treatment	4.1 3.7–4.3	3.9 3.6–4.1	4.5 4.0–4.6
Post 1. treatment	4.3 4.1–4.8	3.5 3.6–4.1	–
24 h after 1. treatment	4.4 3.9–4.6	4.1 3.4–4.3	–
Pre 3. treatment	4.2 3.9–4.4	3.9 3.8–4.3	–
Post 3. treatment	4.1 3.9–4.4	3.4 3.1–3.6	–
Pre 5. treatment	4.4 4.0–4.6	3.5 3.9–4.1	–
Post 5. treatment	4.3 4.0–4.6	3.3 3.1–3.6	–

Table shows serum albumin concentration (g/dl), median and Interquartile range (IQR).

**Figure 1:**
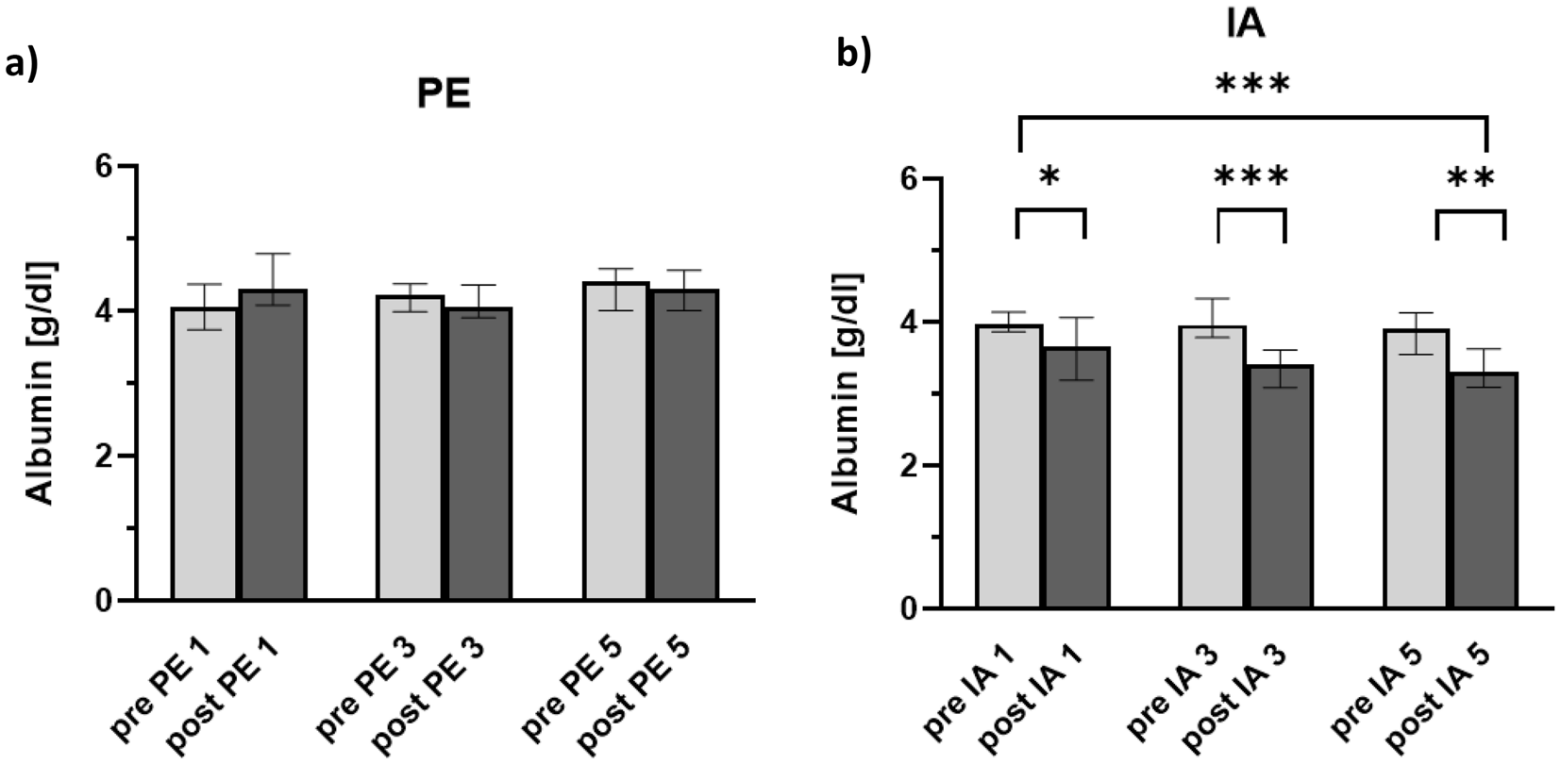
Albumin quantity over the course of therapeutic apheresis. Graphs show median albumin quantity over the course of therapeutic apheresis. Data include (**a**) 20 patients treated with plasmapheresis (PE) and (**b**) 13 patients treated with immunoadsorption (IA). Error bars show interquartile range. Asterisks mark statistically significant differences between times of measurement: Wilcoxon matched-pairs signed rank test, *, *P* < 0.05; **, *P* < 0.01; ***, *P* < 0.001; 2-way ANOVA F (5, 60) = 9.4; *P* < 0.001.

In contrast, albumin level decreased significantly after each IA. After five treatments the albumin level was approximately 15% lower than at the beginning of the IA course and was below the normal range. Detailed information about albumin quantity is presented in Table 3 and Fig. 1 and the supplement Table 3.

### Albumin redox state

Albumin redox state was determined before and after the first, third, and fifth apheresis treatment and also 24 h after the first treatment. The albumin fractions of the commercial albumin used for PE were also determined.

**Figure 2:**
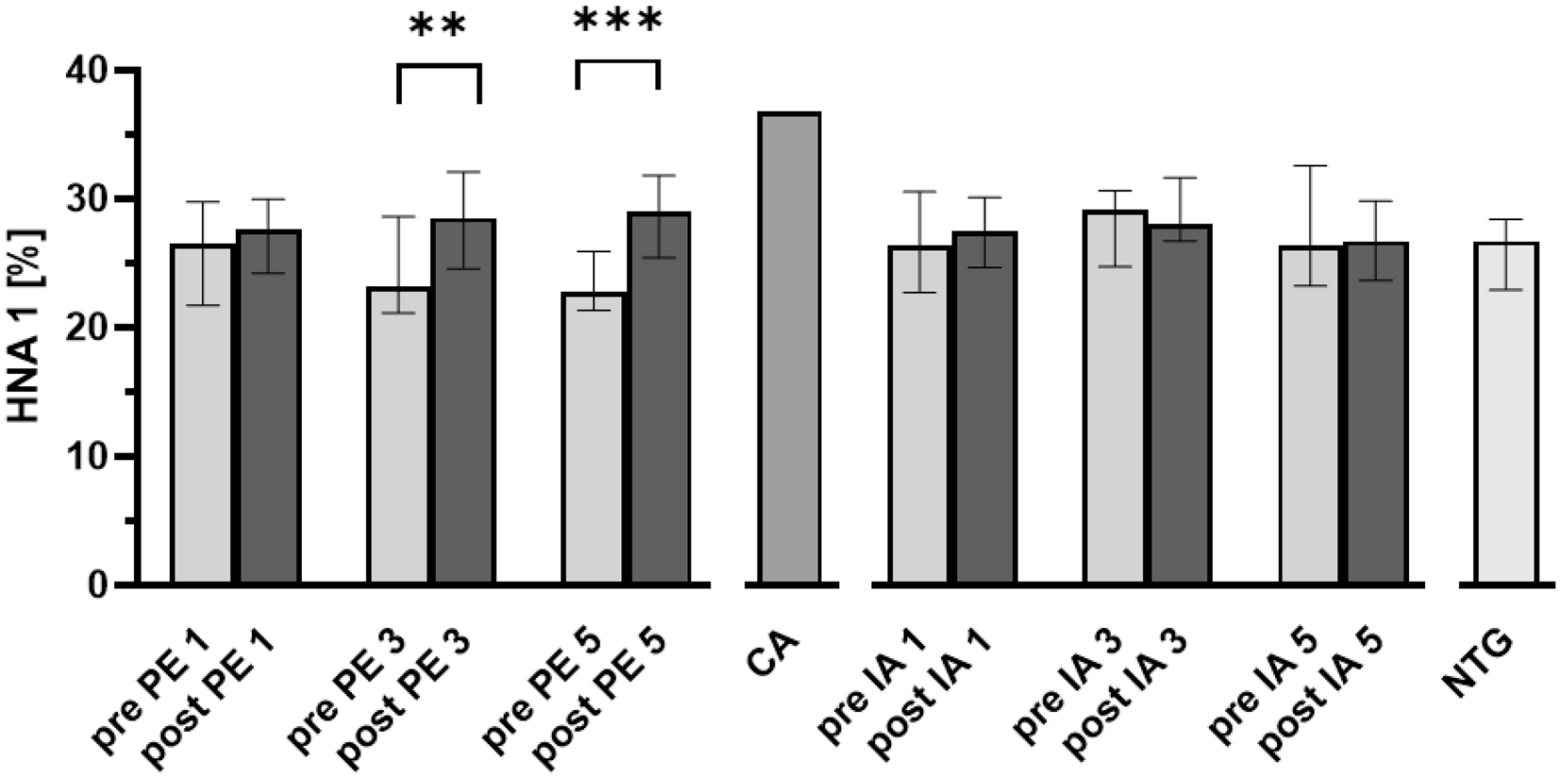
Human nonmercaptalbumin-1 fraction over the course of therapeutic apheresis. Graphs show median human nonmercaptalbumin-1 (HNA-1) fractions over the course of therapeutic apheresis. Data include 20 patients treated with plasmapheresis (PE), 13 patients treated with immunoadsorption (IA), and 10 non-treated patients (NTG). Error bars show interquartile range. CA, commercial albumin solution, Paired t-test, **, *P* < 0.01; ***, *P* < 0.001; 2-way ANOVA F (5, 95) = 7.6; *P* < 0.001.

Before the first treatment there was no evidence of an increased level of oxidative stress, the albumin redox state did not differ between the groups and was in the range of healthy subjects (Table 4^10^). In the PE group, there was an increase of the HNA-1 fraction after each treatment. This was statistically significant after the third and the fifth treatment (*P* < 0.01, *P* < 0.001, respectively). However, with 29.0% HNA-1 it remained within the range of healthy subjects. In the IA group, the HNA-1 fraction remained largely constant over the course of treatment (Fig. 2).

The IA group started with a mean HNA-2 proportion of 3.0%. This increased significantly after the first IA treatment (*P* < 0.05). Also, there was a significant increase in the mean HNA-2 fraction over the course of the five immunoadsorption treatments (*P* < 0.05), but these higher levels were still within the range of healthy subjects (< 5%).

**Figure 3:**
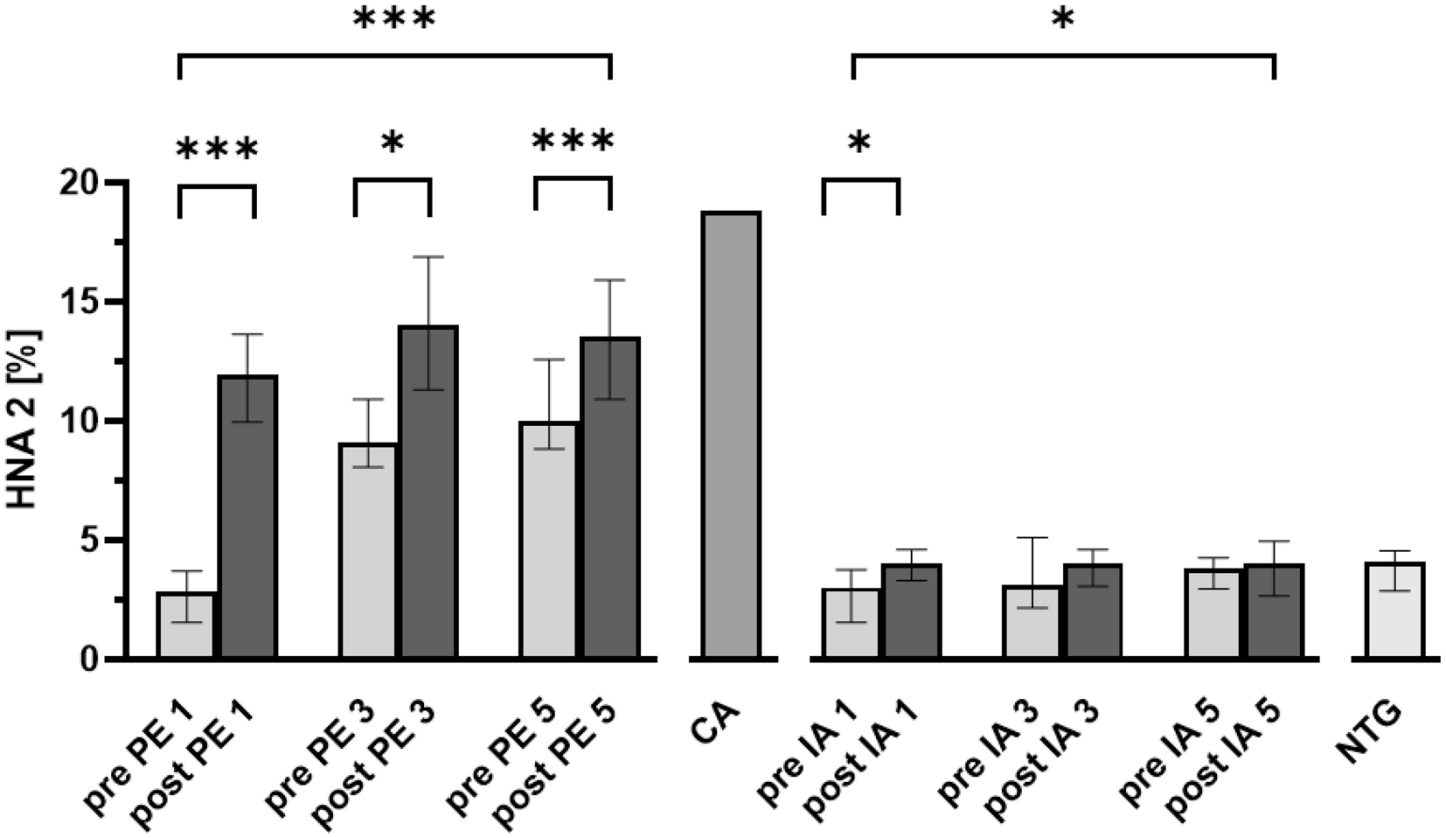
Human nonmercaptalbumin-2 fraction over the course of therapeutic apheresis. Graphs show median human nonmercaptalbumin-2 (HNA-2) fractions over the course of therapeutic apheresis. Data include 20 patients treated with plasmapheresis (PE), 13 patients treated with immunoadsorption (IA), and 10 non-treated patients (NTG). Error bars show interquartile range. CA, commercial albumin solution; for PE-group: paired t-test, *, *P* < 0.05; **, *P* < 0.01; ***, *P* < 0.001; 2-way ANOVA F (5, 95) = 36.3; *P* < 0.001; for IA-group: Wilcoxon matched-pairs signed rank test, *, *P* < 0.05; F (5, 60) = 3.4; *P* = 0.008.

Before the first treatment the HNA-2 proportion was 2.8% in the PE group. After the first treatment there was a pronounced increase in the mean HNA-2 proportion, to 11.9% (*P* < 0.001). At 24 h after the first plasmapheresis treatment, a slight decrease in the HNA-2 level was observed, but over the course of the further treatments the HNA-2 level remained higher than 10%, which corresponds to four times the baseline level (Fig. 3).

**Figure 4:**
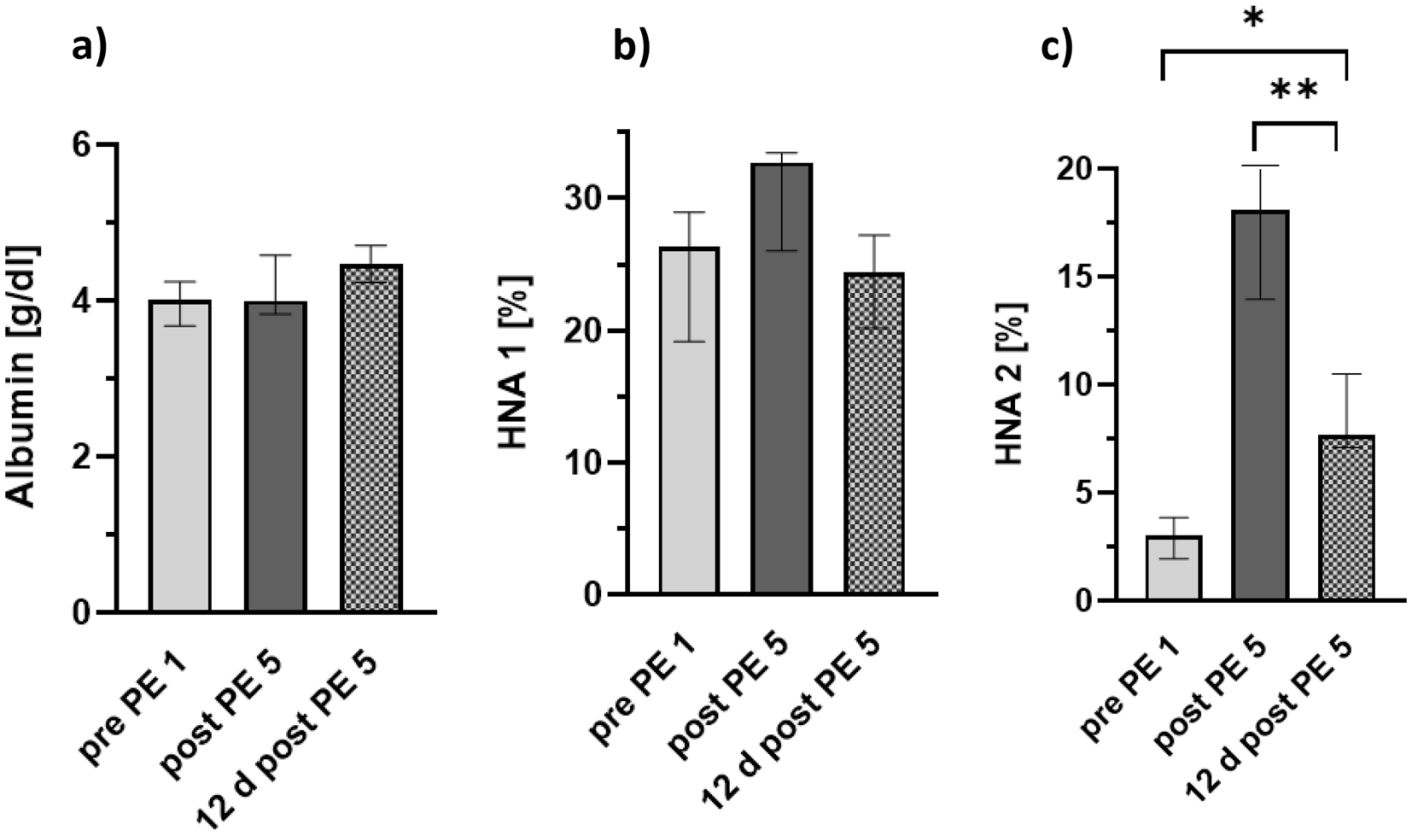
Long-lasting effects on albumin quantity and Human nonmercaptalbumin fractions over the course of plasmapheresis. Graphs show (**a**) median albumin quantity (**b**) Human nonmercaptalbumin-1 fraction (HNA 1) and (**c**) Human nonmercaptalbumin-2 (HNA 2) fraction over the course of plasmapheresis (PE). Data include n = 5 patients. Error bars correspond to interquartile range. Asterisks mark differences between times of measurement, paired t-test, *, *P* < 0.05; ** *P* < 0.01; 2-way ANOVA F (2, 8) = 44.7; *P* < 0.001.

The commercial albumin used for the PE substitution solution showed a HMA proportion of 44.5%, a HNA-1 proportion of 36.8%, and a HNA-2 proportion of 18.8%. This was measured in the diluted albumin solution, which was used for substitution. Detailed information about albumin fractions is presented in Table 4 and Figs. 2 and 3 and the supplement Table 4. Information about the individual course of albumin quantity and albumin fractions are provided in the supplementary section (Supplementary Table 1, Figs. 1–4).

**Table 4:** Albumin fractions.

	HMA			HNA 1			HNA 2		
	Treatment subjects	Control subjects		Treatment subjects	Control subjects		Treatment subjects	Control subjects	
	PE	IA	NTG	PE	IA	NTG	PE	IA	NTG
Pre 1. treatment	72.2 67.3–76.1	70.9 67.3–73.6	71.2 67.5–73.9	26.5 21.7–29.8	26.4 22.7–30.6	25.3 22.9–27.6	2.8 1.3–3.7	3.0 1.6–3.8	4.0 2.8–4.5
Post 1. treatment	60.9 57.3–64.9	68.2 64.8–71.1	–	27.6 24.2–29.9	27.5 24.7–30.1	–	11.9 9.5–13.6	4.0 3.3–4.6	–
24 h after 1. treatment	67.4 63.4–71.7	69.3 63.7–71.6	–	23.1 20.9–26.7	27.9 25.0–32.4	–	8.1 6.3–10.5	4 3.4–4.5	–
Pre 3. treatment	66.2 61.6–70.9	67.2 65.3–69.4	–	22.8 21.1–28.0	29.1 24.7–30.6	–	8.9 8.0–10.9	3.1 2.2–5.1	–
Post 3. treatment	57.3 53.5–63.8	67.5 62.9–70.2	–	28.5 24.6–32.1	28.0 26.7–31.7	–	14.0 11.3–16.9	4.0 3.1–4.6	–
Pre 5. treatment	66.1 63.5–68.8	69.5 62.4–71.8	–	22.8 21.3–25.9	26.4 23.2–32.6	–	9.9 8.5–12.6	3.8 2.9–4.3	–
Post 5. treatment	59.0 54.5–61.0	69.7 64.3–72.0	–	29.0 25.4–31.8	26.7 23.7–29.8	–	13.6 10.9–15.9	4.0 2.7–4.9	–

Table shows albumin fractions according to redox state (%), median and interquartile range (IQR).

*PE* Plasmapheresis, *IA* Immunoadsorption, *NTG* Non-treatment group, *HMA* Human mercaptalbumin, *HNA-1* Human nonmercaptalbumin-1, *HNA-2* Human nonmercaptalbumin-2.

### Long-lasting effects of PE on albumin quantity and albumin redox state

In 5 out of 20 PE patients, albumin quantity and albumin redox state were additionally determined 12 days after the last plasmapheresis. Due to loss-of-follow up, there was no sample at this point of time from the other 15 patients of this study group. The albumin level in this subgroup of patients was also largely constant over the course of the five PE treatments and remained in the normal range 12 days after the last PE.

In these five patients, the median HNA-1-fraction was 26.3% (IQR 19.2–28.9%) prior to treatment, 32.7% (IQR 26.0–33.5%) after the fifth PE-treatment and 24.4% (IQR 20.2–27.2%) 12 days after the fifth treatment. These differences were not statistically significant. On the other hand, HNA-2 fraction before the first plasmapheresis was within the normal range. After five plasmapheresis treatments the HNA-2 fraction distinctly increased up to 18.1% (IQR 13.9–21.4%). This effect was as pronounced as in the total group of 20 patients. 12 days after the last plasmapheresis, the HNA-2 fraction was still significantly increased with a median percentage of 7.7% (IQR 7.1–10.5%). Detailed information about the long-lasting effects on albumin quantity and albumin redox state in these five patients are presented in Fig. 4.

## Discussion

### Key findings

This study evaluated the effects of PE on albumin quantity and albumin redox state in terms of HMA-, HNA-1- and HNA-2-fractions. The albumin quantity remained largely constant over five PE treatments. Before the first PE the albumin redox state corresponded to that of healthy subjects. This was also the case for the patients who served as control subjects (IA and no TA). After the first PE, there was a significant increase in HNA-2 corresponding to four times the baseline HNA-2 level. HNA-2 levels increased steadily over the course of five PE treatments and were still significantly elevated above the normal range 12 days after the last PE. In patients who underwent IA, the proportion of HNA-2 increased over the five treatments within the normal range.

### Comparison with previous studies and future prospects

Several studies evaluated the albumin quantity and the albumin redox state associated with the oxidative stress level in various research areas of medicine, especially in the context of liver cirrhosis, acute kidney injury and sepsis. Hypoalbuminemia is associated with an increased mortality in these patients. Some beneficial effects of commercial albumin infusions have been observed in patients with liver cirrhosis and subsequent ascites, in the context of other diseases this effect could not be clearly demonstrated^20–24^. In neurosciences, albumin quantity has mainly been investigated as a measure of oxidative stress at various levels of disease activity^25^. To our knowledge, this is the first study that longitudinally analyses the effect of PE on albumin quantity and redox state. It was observed earlier, that patients with autoimmune mediated neurological conditions, in particular multiple sclerosis, sometimes experience hypoalbuminemia^26^. However, cohorts without hypoalbumemia have also been reported and the role of albumin in multiple sclerosis is controversially discussed in the literature^14,27^. No instances of hypoalbuminemia were observed in our study and all included patients exhibited serum albumin levels within the normal range before the first apheresis treatment.

Moreover, little is known about the albumin redox state of patients with neurological disorders. Greilberger et al. were the first to evaluate oxidized albumin as a marker of oxidative stress in patients with Alzheimer’s disease^28^. Their results were confirmed and expanded by Costa et al., who evaluated the albumin redox state of plasma and cerebrospinal fluid from patients with Alzheimer’s disease^29^. They found that the levels of albumin oxidation in these patients were much higher than those in healthy control subjects and were more pronounced in the cerebrospinal fluid than in plasma.

Our study is the first to analyse the albumin redox state in these patients in general and in particular over the course of plasmapheresis. Unlike reported for Alzheimer’s disease, there was no evidence of an increased level of oxidative stress in both the PE group and the control groups (IA and no TA), since the HNA-1 and the HNA-2 proportions were within the range of healthy subjects before the first apheresis treatment.

The decrease of albumin concentrations during IA treatment is remarkable. A mean decrease of about 4.7 g/L means a considerable amount of albumin loss during one treatment, adsorbed to the apheresis column. Furthermore, although it remained in the range of healthy subjects (< 5%), the proportion of HNA-2 increased significantly over five IA treatments. Because commercial albumin was not administered during IA treatments, this is an increase in endogenous irreversibly oxidized albumin, and this effect must be due to an oxidative challenge due to the treatment itself. In contrast to HNA-2, the fraction of HNA-1, the reversibly oxidized albumin, remained unchanged during IA treatment. This indicates that, despite the oxidative stress caused by IA treatment indicated by increased HNA-2, the systemic redox-balance of the patients is still intact.

For patients who underwent PE, this study showed for the first time a significant increase of reversibly oxidized HNA1 and irreversibly oxidized albumin HNA-2 after treatment. This is no surprise taking into account the high fractions of HNA-1 (36.8%) and HNA-2 (18.8%) in the infusion solution exchanging a considerable proportion of endogenous albumin. Thereby HNA-1 in the infusion solution amounts 144% and HNA-2 about 750% of the mean fraction in patients’ albumin before treatment, respectively. Commercial albumin solutions mainly show a high proportion of oxidised albumin due to the manufacturing process. The stabilizers used there may have an impact on the redox potential, as well as on the binding capacity of the albumin^12^. The increase of HNA-1 was observed only directly after treatment while 12 d post treatment the initial levels were reached again. In contrast, HNA-2 was increased even 12 d post treatment. This is presumably due to the fact, that HNA-1 may be endogenously reduced to HMA, which is a quick reaction, while HNA-2 may only be removed by degradation.

Evaluation of the effects observed in this study with regard to the consequences for the treatment of patients with other indications for PE must take the following considerations into account: The results do not reflect necessarily the albumin redox state in general in a patient. During PE treatment, the patient's plasma is not first completely removed and then completely replaced by an albumin solution, but removal and infusion are continuously. Additionally, only about 40% of the albumin is found in the intravascular space and there is a continuous exchange between the intra- and extravascular albumin space. As a consequence, blood samples drawn from the patients always contain a mixture of the patient’s own albumin and the infused commercial albumin^10,14^.

Furthermore, although HNA-2 has been identified as a risk factor in liver disease^11^, it has not yet been sufficiently investigated whether the high HNA-2 proportion caused by commercial albumin constitutes an equally large amount of damage due to oxidative stress, as HNA-2 produced by the body itself. This addresses the question whether an increase in oxidized albumin fractions is merely a consequence of an oxidative challenge or in addition a cause of further pathologies, which would mean for the patients studied here an addition of further damage by fostering the inflammatory destruction caused by the underlying condition, which should be treated by TA. This question is yet to be answered. Future studies must also clarify whether a high level of HNA-2 due to a PE treatment is associated with a decrease in the albumin function and whether its high quantity exerts a direct harmful influence.

On top of that, one has to take into account another issue of this context: if it seems possible or cannot be excluded according to the current state of knowledge, that the infusion of large amounts of commercial albumin, with a high proportion of irreversibly oxidized HNA 2, may lead to an increase of the oxidative stress level, such an infusion should be replaced by another modified commercial albumin, e. g. containing less stabilizers like octanoates. Various approaches are currently being investigated to improve the quality of commercial albumin before infusion with regard to redox state and binding properties^12,30,31^.

In this study, the severely increased HNA-2 proportion was observed among patients who did not have hepatic or renal impairment, or, in other words, in patients in whom we assume that the systemic redox-balance is still intact and who should be able to endogenously reduce or degrade oxidized albumin in a short time. However, most patients who undergo PE for another indication, e.g., liver failure, thrombotic microangiopathy, or acute rejection after solid organ transplantation, do have liver or kidney impairments. Infusion of large amounts of commercial albumin could mean an additional burden in a condition of already acute serious illness. Therefore, the results of this study are of particular relevance for these patients with hepatic or renal impairment and should encourage further improvement of the quality of commercial albumin.

### Strengths and limitations

Our study has several strengths and limitations. To our knowledge, this is the first longitudinal prospective study to evaluate the influence of PE on albumin quantity and albumin redox state in patients with autoimmune mediated neurological condition. For this, two different redox-markers, a reversible and an irreversible oxidation, in the same molecule have been investigated. The study consisted of two different control groups, with and without an extracorporeal therapy. The IA-group served as the first control for any changes of albumin redox state that might occur due to the extracorporeal circuit and the non-treatment group as a second control for a maybe already altered albumin redox state due to the underlying neurological disease. The results are limited by the monocentric study design with a small sample size and needs validation by additional studies with larger patient cohorts. Also, only a part of the PE-study group could be evaluated 12 days after the last procedure due to loss-to-follow up, which may lead to a certain selection bias. One reason for this could have been the often wide distance between patients’ home and the clinic, so the follow-up examination was carried out in an outpatient basis. Patients have not been randomized to the treatment/non-treatment groups. This could lead also to a certain selection bias, as only more severly ill patients received TA. In terms of oxidative stress, albumin redox state is one of the variables, so our results are indicating a new adverse effect of PE but need validation by additional studies with evaluation of other variables (eg. homocysteine, serum and intraerythrocyte folate) for a greater generalizability.

## Conclusion

The present study showed for the first time that plasmapheresis exerts a severe and long-lasting alteration on albumin redox state, which lasted at least 12 days indicating a new adverse effect of PE that may influence oxidative stress level.

## Supplementary Information

Supplementary Information.

## Data Availability

The data presented in this study are available on request from the corresponding author.

## Abbreviations

ACE: Angiotensin-converting enzyme
CIDP: Chronic inflammatory demyelinating polyneuropathy
GBS: Guillain–Barré syndrome
HMA: Human mercaptalbumin
HNA-1: Human non-mercaptalbumin 1
HNA-2: Human non-mercaptalbumin 2
HPLC: High-performance liquid chromatography
IA: Immunoadsorption
PE: Plasmapheresis

## References

[1] Deutsche Gesellschaft für Nephrologie. Apherese-Standard. https://www.dgfn.eu/apherese-standard.html. Accessed 2 Sept 2021.

[2] Padmanabhan A, Connelly-Smith L, Aqui N, Balogun RA, Klingel R, Meyer E, Pham HP, Schneiderman J, Witt V, Wu Y, Zantek ND, Dunbar NM, Schwartz GEJ (2019). Guidelines on the use of therapeutic apheresis in clinical practice—Evidence-based approach from the writing committee of the American Society for Apheresis: The eighth special issue. J. Clin. Apher..

[3] Rajabally YA, Stettner M, Kieseier BC, Hartung HP, Malik RA (2017). CIDP and other inflammatory neuropathies in diabetes—Diagnosis and management. Nat. Rev. Neurol..

[4] Davies AJ, Fehmi J, Senel M, Tumani H, Dorst J, Rinaldi S (2020). Immunoadsorption and plasma exchange in seropositive and seronegative immune-mediated neuropathies. J. Clin. Med..

[5] Dorst J, Fangerau T, Taranu D, Eichele P, Dreyhaupt J, Michels S, Schuster J, Ludolph AC, Senel M, Tumani H (2019). Safety and efficacy of immunoadsorption versus plasma exchange in steroid-refractory relapse of multiple sclerosis and clinically isolated syndrome: A randomised, parallel-group, controlled trial. EClinicalMedicine.

[6] Klingele M, Allmendinger C, Thieme S, Baerens L, Fliser D, Jan B (2020). Therapeutic apheresis within immune-mediated neurological disorders: Dosing and its effectiveness. Sci. Rep..

[7] Lipphardt M, Mühlhausen J, Kitze B, Heigl F, Mauch E, Helms HJ, Müller GA, Koziolek MJ (2019). Immunoadsorption or plasma exchange in steroid-refractory multiple sclerosis and neuromyelitis optica. J. Clin. Apher..

[8] Bramlage CP, Schröder K, Bramlage P, Ahrens K, Zapf A, Müller GA, Koziolek MJ (2009). Predictors of complications in therapeutic plasma exchange. J. Clin. Apher..

[9] Yang M, Liao C, Zhu Q, Lin X, Yang B, Zhao D, Li J, Deng D, Zhang W (2020). Meta-analysis on the efficacy and safety of immunoadsorption for systemic lupus erythematosus among Chinese population. Clin. Rheumatol..

[10] Oettl K, Marsche G (2010). Redox state of human serum albumin in terms of cysteine-34 in health and disease. Methods Enzymol..

[11] Oettl K, Birner-Gruenberger R, Spindelboeck W, Stueger HP, Dorn L, Stadlbauer V, Putz-Bankuti C, Krisper P, Graziadei I, Vogel W, Lackner C, Stauber RE (2013). Oxidative albumin damage in chronic liver failure: Relation to albumin binding capacity, liver dysfunction and survival. J. Hepatol..

[12] Farrugia A, Mori F (2022). Therapeutic solutions of human albumin—The possible effect of process-induced molecular alterations on clinical efficacy and safety. J. Pharm. Sci..

[13] Fanali G, di Masi A, Trezza V, Marino M, Fasano M, Ascenzi P (2012). Human serum albumin: From bench to bedside. Mol. Asp. Med..

[14] LeVine SM (2016). Albumin and multiple sclerosis. BMC Neurol..

[15] Roche M, Rondeau P, Singh NR, Tarnus E, Bourdon E (2008). The antioxidant properties of serum albumin. FEBS Lett..

[16] Cholongitas E, Papatheodoridis GV, Vangeli M, Terreni N, Patch D, Burroughs AK (2005). Systematic review: The model for end-stage liver disease–should it replace Child–Pugh’s classification for assessing prognosis in cirrhosis?. Aliment. Pharmacol. Ther..

[17] O’Grady JG, Alexander GJ, Hayllar KM, Williams R (1989). Early indicators of prognosis in fulminant hepatic failure. Gastroenterology.

[18] KDIGO clinical practice guideline for acute kidney injury. *Kidney Int. Suppl.* **2**(1), 1–138 (2012). https://kdigo.org/wp-content/uploads/2016/10/KDIGO-2012-AKI-Guideline-English.pdf. Accessed 2 Sept 2021.

[19] KDIGO 2012 clinical practice guideline for the evaluation and management of chronic kidney disease. *Kidney Int. Suppl.* **3**(1), 1–150 (2013). https://kdigo.org/wp-content/uploads/2017/02/KDIGO_2012_CKD_GL.pdf. Accessed 2 Sept 2021. 10.1038/ki.2013.243 23989362

[20] Bernardi M, Angeli P, Claria J, Moreau R, Gines P, Jalan R, Caraceni P, Fernandez J, Gerbes AL, O’Brien AJ, Trebicka J, Thevenot T, Arroyo V (2020). Albumin in decompensated cirrhosis: New concepts and perspectives. Gut.

[21] Figueroa SM, Araos P, Reyes J, Gravez B, Barrera-Chimal J, Amador CA (2021). Oxidized albumin as a mediator of kidney disease. Antioxidants (Basel)..

[22] Wiedermann CJ, Wiedermann W, Joannidis M (2010). Hypoalbuminemia and acute kidney injury: A meta-analysis of observational clinical studies. Intensive Care Med..

[23] Vincent JL, De Backer D, Wiedermann CJ (2016). Fluid management in sepsis: The potential beneficial effects of albumin. J. Crit. Care.

[24] Colombo G, Clerici M, Giustarini D, Rossi R, Milzani A, Dalle-Donne I (2012). Redox albuminomics: Oxidized albumin in human diseases. Antioxid. Redox Signal..

[25] Zhang SY, Gui LN, Liu YY, Shi S, Cheng Y (2020). Oxidative stress marker aberrations in multiple sclerosis: A meta-analysis study. Front. Neurosci..

[26] Oliveira SR, Kallaur AP, Reiche EMV, Kaimen-Maciel DR, Panis C, Lozovoy MAB, Morimoto HK, Maes M, Dichi I, Simão ANC (2017). Albumin and protein oxidation are predictors that differentiate relapsing-remitting from progressive clinical forms of multiple sclerosis. Mol. Neurobiol..

[27] Xue H, Yang Z, Wang L, Jiang Y, Li J, Wu M, Wang G, Zhang Y, Zhang M (2021). Factors influencing the degree of disability in patients with multiple sclerosis. Front. Neurol..

[28] Greilberger J, Koidl C, Greilberger M, Lamprecht M, Schroecksnadel K, Leblhuber F, Fuchs D, Oettl K (2008). Malondialdehyde, carbonyl proteins and albumin-disulphide as useful oxidative markers in mild cognitive impairment and Alzheimer’s disease. Free Radic. Res..

[29] Costa M, Horrillo R, Ortiz AM, Pérez A, Mestre A, Ruiz A, Boada M, Grancha S (2018). Increased albumin oxidation in cerebrospinal fluid and plasma from Alzheimer’s disease patients. J. Alzheimers Dis..

[30] Oettl K, Stadlbauer V, Krisper P, Stauber RE (2009). Effect of extracorporeal liver support by molecular adsorbents recirculating system and Prometheus on redox state of albumin in acute-on-chronic liver failure. Ther. Apher. Dial..

[31] Mikkat S, Dominik A, Stange J, Eggert M (2020). Comparison of accompanying proteins in different therapeutic human serum albumin preparations. Biologicals.

