# Supplementary material for: Severe and long-lasting alteration of albumin redox state by plasmapheresis
Source: Sci Rep. 2022 Jul 16;12:12165. doi: 10.1038/s41598-022-16452-4 (PMC9288533; doi:10.1038/s41598-022-16452-4)
Supplement: Supplementary file 1 — Supplementary Information. [file 41598_2022_16452_MOESM1_ESM.docx]

**Severe and long-lasting alteration of albumin redox state**

**by plasmapheresis**

Kristina Boss^1^, Mark Stettner^2^, Fabian Szepanowski^2^, Anne K Mausberg^2^, Margret Paar^3^, Refik Pul^2^, Christoph Kleinschnitz^2^, Karl Oettl^3*^ and Andreas Kribben^1*^

^1^ Department of Nephrology, University Hospital Essen, University Duisburg-Essen, Essen,

Germany

^2^ Department of Neurology and Center for Translational Neuro- and Behavioural Sciences (C-

TNBS), University Hospital Essen, University Duisburg-Essen, Essen, Germany

^3^ Division of Physiological Chemistry, Otto Loewi Research Center, Medical University of

Graz, Graz, Austria

*These authors contributed equally to the manuscript.

**Supplements**

**PE**


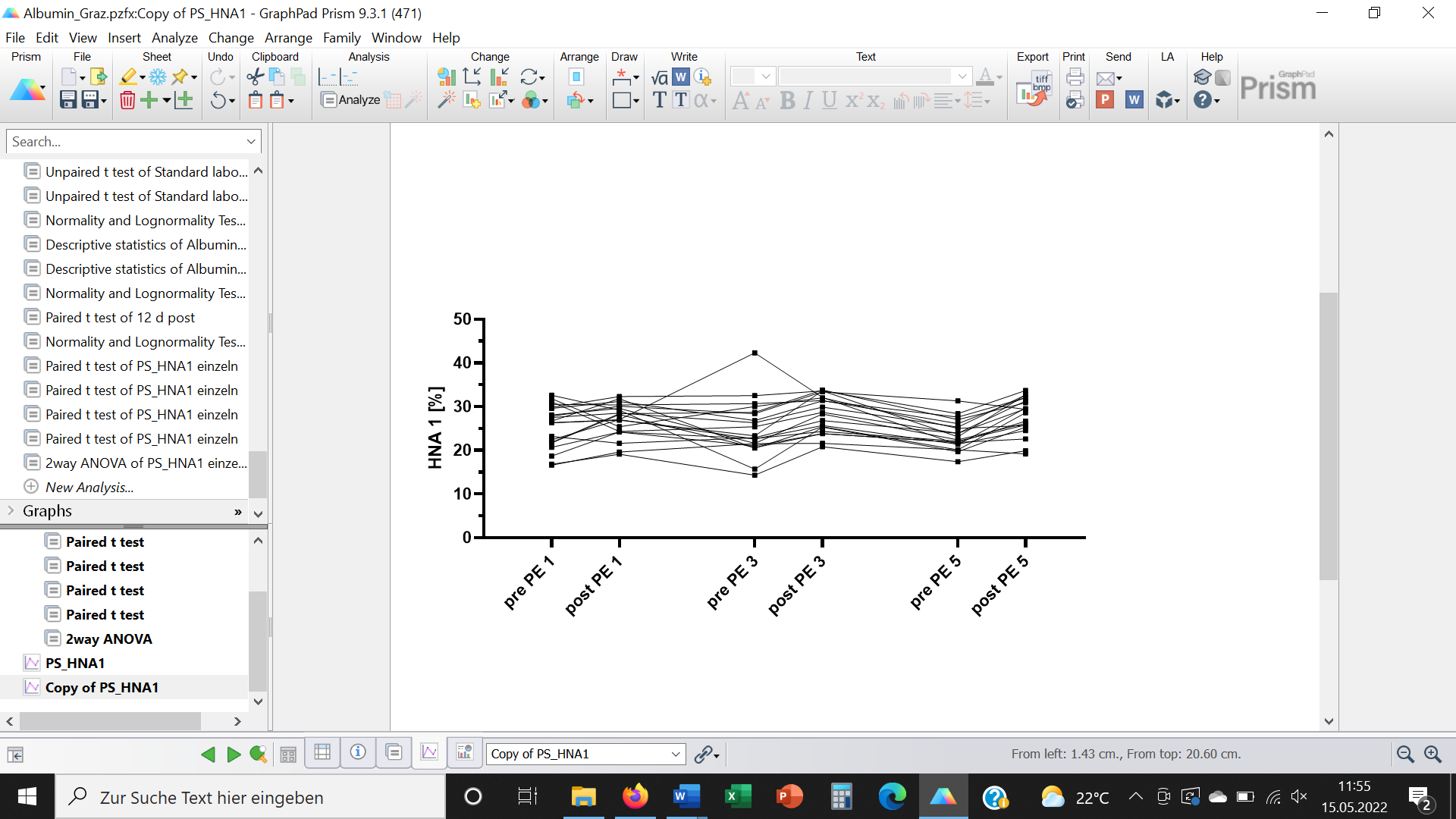


**Figure 1: Individual course of Human nonmercaptalbumin-1 fraction in 20 subjects over five PE treatments**

Graphs show individual course of human nonmercaptalbumin-1 (HNA-1) fractions over five plasmapheresis (PE) treatments. Data include 20 patients.

**IA**


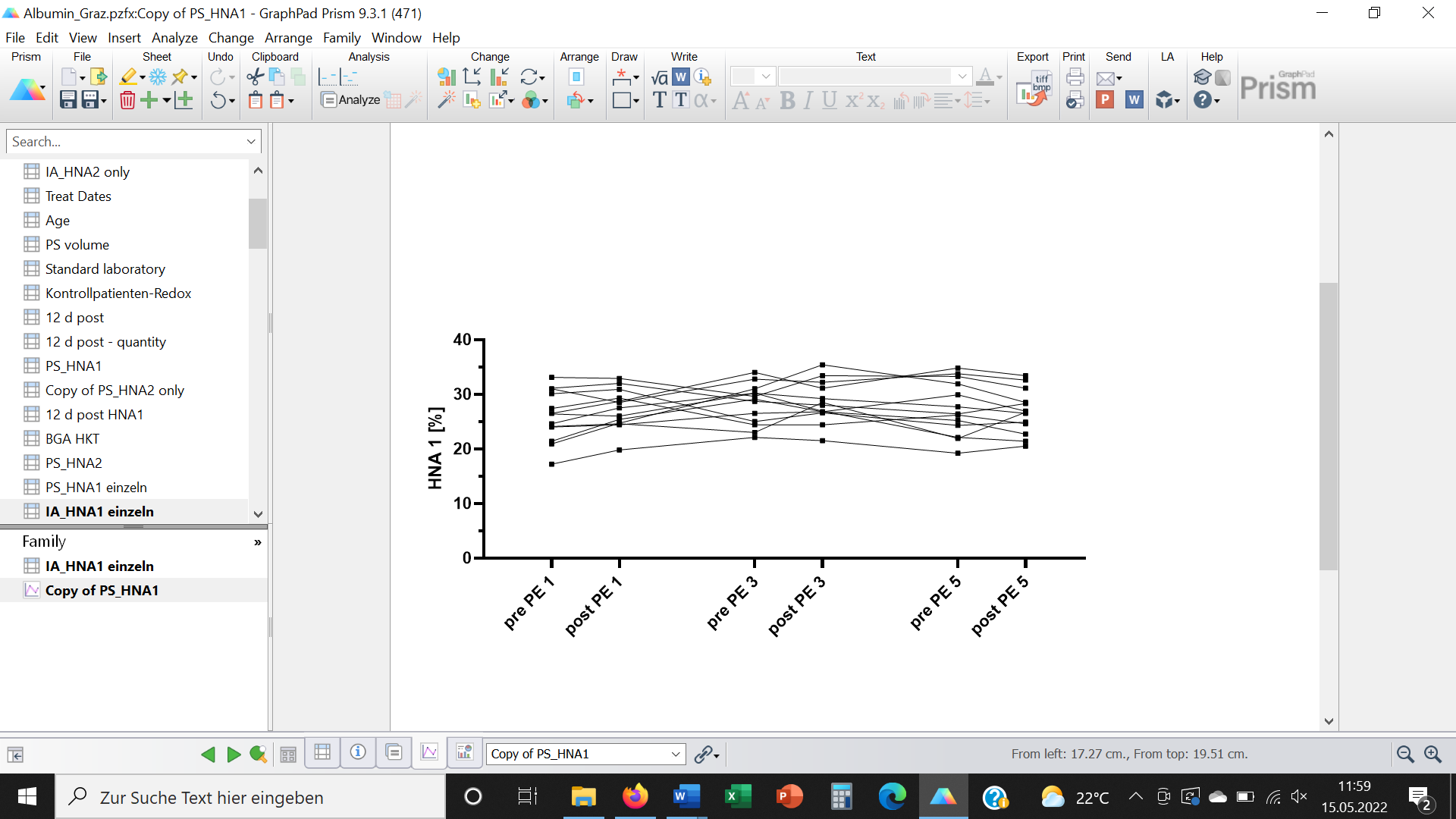


**Figure 2: Individual course of Human nonmercaptalbumin-1 fraction in 13 subjects over five IA treatments**

Graphs show individual course of human nonmercaptalbumin-1 (HNA-1) fractions over five immunoadsorption (IA) treatments. Data include 13 patients.


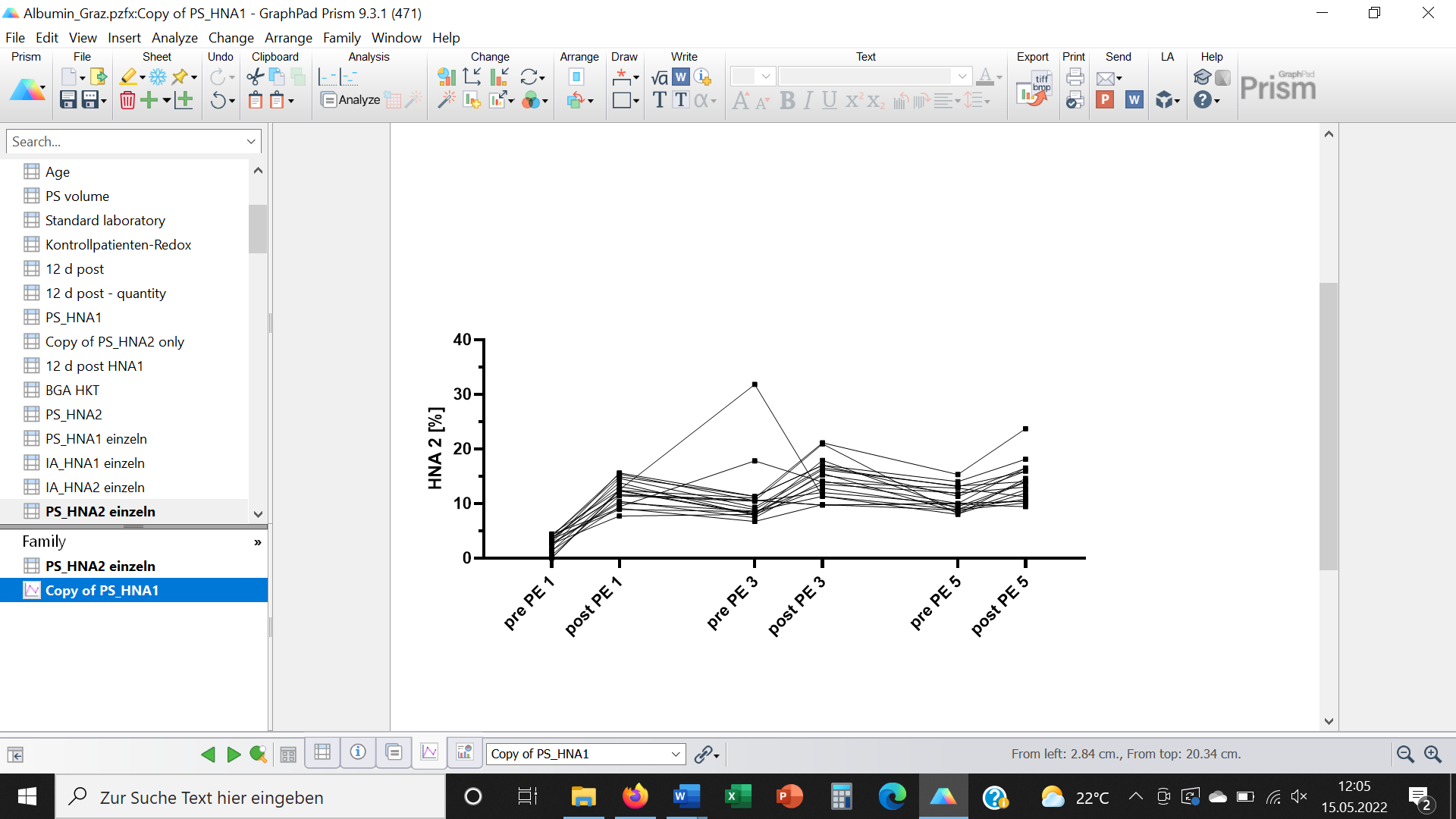


**PE**

**Figure 3: Individual course of Human nonmercaptalbumin-2 fraction in 20 subjects over five PE treatments**

Graphs show individual course of human nonmercaptalbumin-2 (HNA-2) fractions over five plasmapheresis (PE) treatments. Data include 20 patients.


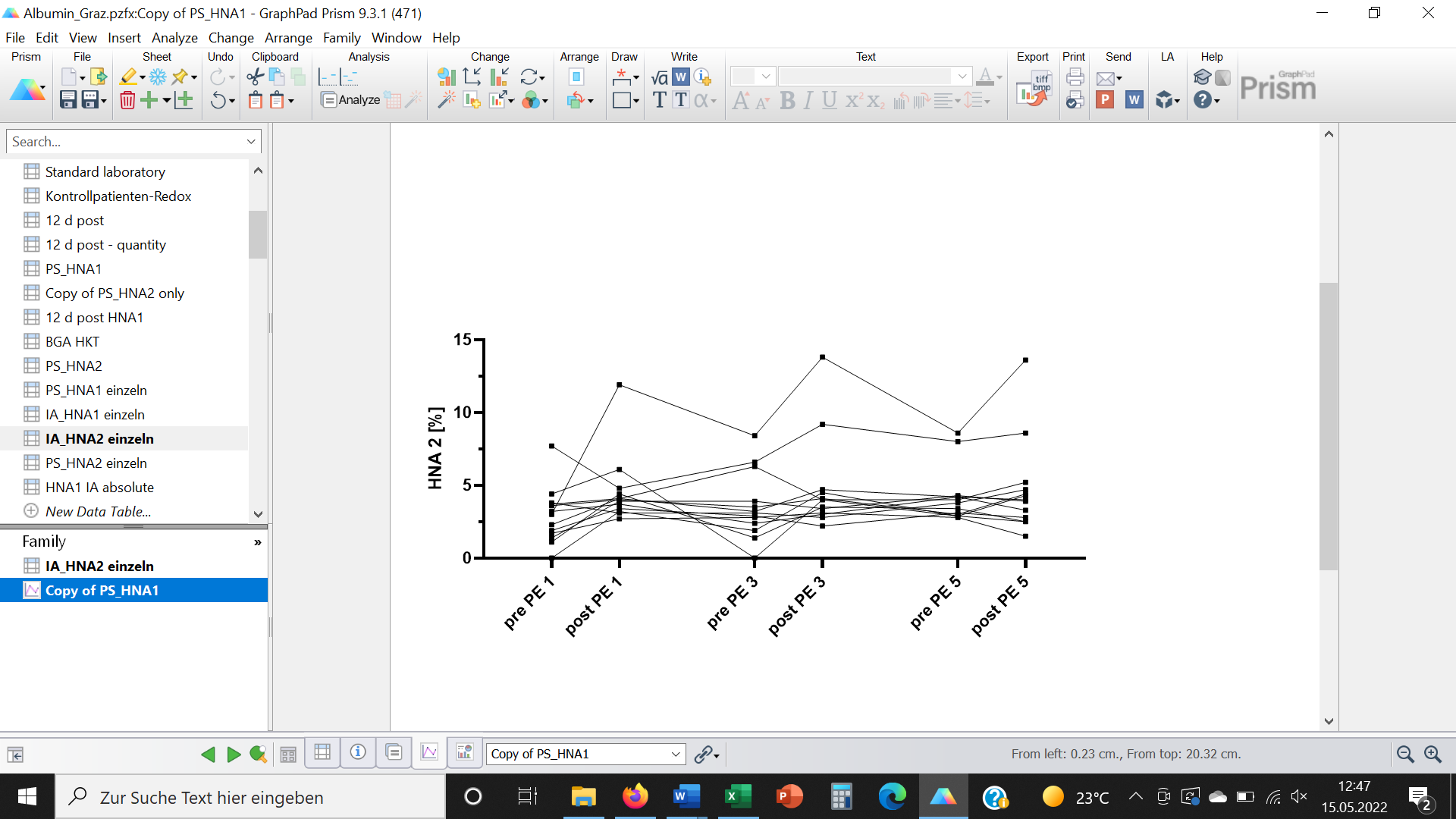


**IA**

**Figure 4: Individual course of Human nonmercaptalbumin-2 fraction in 13 subjects over five IA treatments**

Graphs show individual course of human nonmercaptalbumin-2 (HNA-2) fractions over five immunoadsorption (IA) treatments. Data include 13 patients.

**Table 1: Albumin quantity and albumin fractions**

|  | Albumin quantity [g/dl] | HNA 1  [%] | HNA 1  [g/dl] | HNA 2  [%] | HNA 2  [g/dl] |
| --- | --- | --- | --- | --- | --- |
| Pre  1.treatment | 3.9  3.6-4.1 | 26.4  22.7-30.6 | 1.0  0.8-1.3 | 3.0  1.6-3.8 | 0.1  0.1-0.1 |
| Post  1.treatment | 3.5  3.6-4.1 | 27.5  24.7-30.1 | 1.0  0.8-1.1 | 4.0  3.3-4.6 | 0.1  0.1-0.2 |
| 24 h after  1.treatment | 4.1  3.4-4.3 | 27.9  25.0-32.4 | 1.0  0.8-1.1 | 4  3.4-4.5 | 0.1  0.1-0.2 |
| Pre  3.treatment | 3.9  3.8-4.3 | 29.1  24.7-30.6 | 1.2  0.9-1.3 | 3.1  2.2-5.1 | 0.1  0.1-0.2 |
| Post  3.treatment | 3.4  3.1-3.6 | 28.0  26.7-31.7 | 0.9  0.8-1.1 | 4.0  3.1-4.6 | 0.1  0.1-0.1 |
| Pre  5.treatment | 3.5  3.9-4.1 | 26.4  23.2-32.6 | 1.0  0.9-1.2 | 3.8  2.9-4.3 | 0.1  0.1-0.2 |
| Post  5.treatment | 3.3  3.1-3.6 | 26.7  23.7-29.8 | 0.9  0.7-1.0 | 4.0  2.7-4.9 | 0.1  0.1-0.2 |

*Table shows albumin quantity and albumin fractions according to redox state [%] and as absolute concentrations [g/dl] as median and interquartile range (IQR); IA, immunoadsorption; HMA, human mercaptalbumin; HNA-1, human nonmercaptalbumin-1; HNA-2, human nonmercaptalbumin-2.*

**Table 2 Additional statistics on patient characteristics and baseline measurements**

|  | Normally distributed in all groups according to Kolmogorov-Smirnov-Test? | PE vs. IA | PE vs. NTG | IA vs. NTG |
| --- | --- | --- | --- | --- |
| Age | yes | p = 0.11 | p = 0.64 | p = 0.06 |
| White blood cell count | yes | p = 0.78 | p = 0.52 | p = 0.10 |
| Haemoglobin | yes | p = 0.35 | p = 0.06 | p = 0.05 |
| Haematocrit | yes | p = 0.24 | p = 0.07 | p = 0.06 |
| Platelets | yes | p = 0.55 | p = 0.07 | p = 0.07 |
| Albumin | yes | p = 0.47 | p = 0.37 | p = 0.29 |
| C-reactive protein | yes | p = 0.52 | p = 0.73 | p = 0.77 |
| Fibrinogen | yes | p = 0.34 | p = 0.26 | p = 0.14 |

*Table shows significance testing between treatment group and control groups, unpaired t-test with p < 0.05; PE, plasmapheresis; IA, immunoadsorption; NTG, non-treatment group*

**Table 3 Additional statistics on albumin quantity**

|  | Treatment subjects | Control subjects | |
| --- | --- | --- | --- |
|  | Plasmapheresis | Immunoadsorption | Non-treatment Group |
|  | n = 20 | n = 13 | n = 10 |
| Pre 1. treatment | 4.1  3.7-4.3  *4.1 ± 0.6* | 3.9  3.6-4.1  - | 4.5  4.0-4.6  *4.5 ± 0.3* |
| Post 1. treatment | 4.3  4.1-4.8  *4.4 ± 0.4* | 3.5  3.6-4.1  *3.5 ± 0.7* | - |
| 24 h after 1. treatment | 4.4  3.9-4.6  *4.4 ± 0.6* | 4.1  3.4-4.3  *4.1 ± 0.5* | - |
| Pre 3. treatment | 4.2  3.9-4.4  *4.2 ± 0.5* | 3.9  3.8-4.3  - | - |
| Post 3. treatment | 4.1  3.9-4.4  *4.1 ± 0.4* | 3.4  3.1-3.6  *3.3 ± 0.5* | - |
| Pre 5. treatment | 4.4  4.0-4.6  *4.3 ± 0.5* | 3.5  3.9-4.1  *3.7 ± 0.7* | - |
| Post 5. treatment | 4.3  4.0-4.6  *4.3 ± 0.4* | 3.3  3.1-3.6  *3.3 ± 0.5* | - |

Table shows serum albumin concentration [g/dl], median and Interquartile range (IQR), in italics mean ± SD if data is normally distributed according to Kolmogorov-Smirnov-Test.

**Table 4 Additional statistics on albumin fractions**

|  | HMA | | | HNA 1 | | | HNA 2 | | |
| --- | --- | --- | --- | --- | --- | --- | --- | --- | --- |
|  | Treatment subjects | Control subjects | | Treatment subjects | Control subjects | | Treatment subjects | Control subjects | |
|  | PE | IA | NTG | PE | IA | NTG | PE | IA | NTG |
| Pre  1.treatment | 71.9 ± 5.3 | 71.1 ± 5.2 | 70.9 ± 3.8 | 25.5 ± 4.9 | 25.9 ± 4.6 | 25.3 ± 3.1 | 2.5 ± 1.4 | 2.9 ± 1.9 | 3.8 ± 0.9 |
| Post  1.treatment | 61.6 ± 5.9 | 68.1 ± 3.9 | - | 26.9 ± 3.8 | 27.3 ± 3.6 | - | 11.4 ± 3.1 | 4.6 ± 2.3 | - |
| 24 h after  1.treatment | 67.1 ± 7.3 | 68.3 ± 4.0 | - | 23.6 ± 4.7 | 27.6 ± 4.4 | - | 9.3 ± 5.7 | 4.2 ± 1.8 | - |
| Pre  3.treatment | 65.2 ± 9.2 | 68.2 ± 3.9 | - | 24.5 ± 6.2 | 28.2 ± 3.7 | - | 10.4 ± 5.8 | 3.6 ± 2.3 | - |
| Post  3.treatment | 57.8 ± 7.5 | 66.7 ± 4.9 | - | 28.3 ± 4.3 | 28.5 ± 3.8 | - | 13.9 ± 4.2 | 4.8 ± 3.2 | - |
| Pre  5.treatment | 66.3 ± 4.5 | 68.3 ± 5.3 | - | 23.5 ± 3.4 | 27.4 ± 4.9 | - | 10.2 ± 2.9 | 4.2 ± 1.9 | - |
| Post  5.treatment | 58.7 ± 6.9 | 68.5 ± 5.2 | - | 27.9 ± 4.2 | 26.8 ± 4.0 | - | 13.4 ± 4.2 | 4.7 ± 3.2 | - |

*Table shows albumin fractions according to redox state [%], mean ± SD. PE, plasmapheresis; IA, immunoadsorption; NTG, non-treatment group; HMA, human mercaptalbumin; HNA-1, human nonmercaptalbumin-1; HNA-2, human nonmercaptalbumin-2.*
